# Hurdles to open access publishing faced by authors: a scoping literature review from 2004 to 2023

**DOI:** 10.1098/rsos.250257

**Published:** 2025-08-20

**Authors:** Nataliia Kaliuzhna, Zeynep Aydin, Paul Müller, Christian Hauschke

**Affiliations:** ^1^ Lab Group Open Research Information, TIB Leibniz Information Centre for Science and Technology, Hanover, Germany; ^2^ Information Technologies Department, Kyiv National University of Culture and Arts, Kyiv, Ukraine; ^3^ University of Technology Nuremberg, Nuremberg, Germany

**Keywords:** open access barriers, scholarly communication, academic publishing, open science

## Abstract

Over the past two decades, numerous widespread efforts and individual contributions to shift scientific publishing to open access (OA) have faced a number of obstacles. Due to the complexity of knowledge production dimension and knowledge dissemination, the challenges encountered by researchers, publishers and readers differ. While examples of such barriers exist across multiple parties, no attempt has been made to synthesize these for active researchers. Thus, this scoping review explores the barriers documented in the scientific literature that researchers encounter in their pursuit of publishing open access. After screening 1280 relevant sources, 113 papers, published between 2004 and 2023, were included in the review. A total of 82 distinct barriers were identified and grouped into four subclusters: Practical Barriers, Lack of Competency, Sentiment, and Policy and Governance. The largest cluster in terms of barriers assigned was Sentiment, accounting for 51.2% (*n* = 42) of all barriers identified, suggesting that perceived barriers are the strongest determinants of publishing OA, while the most frequently occurring barrier was ‘high article processing charges’, reported in 88 papers. Furthermore, burdens faced specifically due to the location of the researcher were identified. Understanding and acknowledging these barriers are essential for their effective elimination or mitigation.

## 1. Introduction

Publications are the most important instrument for the documentation and dissemination of new, reliable knowledge in science. A large portion of these publications are disseminated in the form of journal articles [1,2]. The publication process involves several steps that include, but are not limited to, conducting the research, writing the manuscript, submission, review and finally publication, which then enables it to be recognized [3]. However, this process can be inflicted with various hurdles that might prevent authors from making their findings available to the public. Potential readers are also confronted with numerous obstacles when they want to study scientific publications. One well-known difficulty that readers face is the paywall for articles, or access to digital journals, which is often restricted to academic libraries. In response to the drastic increase in the price of scientific journals at the turn of the millennium (also known as the journal crisis), the so-called open access (OA) movement developed, which defined its principles and guidelines in various manifestos. Already the first of these documents, the Budapest Open Access Initiative [4], mentioned barriers that had to be removed from scholarly communication. The BOAI mostly tackled the side of access to published science and barriers to publishing were not discussed. The Bethesda Statement on Open Access Publishing [5] already mentioned barriers for researchers, too. It required removing financial disadvantages not only, but especially for researchers from low- and middle-income countries (LMICs). The third of these so-called BBB declarations, the Berlin Declaration [6], did not mention obstacles for researchers again.

Evidently, it was established early on that potential barriers for researchers who wish to publish their research as OA are present. However, a knowledge gap exists in terms of what these barriers are, and there is surprisingly little research on these barriers and who is most affected by them. Although OA publishing is becoming increasingly easy for researchers from rich countries and well-funded research institutions, it is worth taking a look at those who are less fortunate.

Despite the abundance of research discussing barriers to OA publishing, there have been surprisingly few systematic efforts to comprehensively map these hurdles. Xia [7] made an important contribution in this regard by performing a longitudinal study on scholar attitudes and behaviour towards OA publishing. To this end, a statistical time series analysis was conducted on 18 papers published between 1992 and 2009, all of which employed quantitative survey methods. Severin *et al.* [8] carried out a meta-synthesis of 11 bibliometric studies published between 2006 and 2018, analysing both the prevalence of OA publishing across disciplines and the specific barriers that impede researchers’ engagement with OA. Borrego [9] reviewed literature on article processing charges (APCs), examining 168 papers published over two decades, from 2003 to 2023. The review highlights authors’ perspectives on APCs as well as the inequalities they introduce. Azadbakht *et al.* [10] conducted a systematized review of the literature to investigate whether OA mandates and policies increase the rate of OA publishing and data sharing within the affected research communities. This study included 11 papers published between 2011 and 2020.

It is imperative to identify barriers to OA publishing because only by overcoming them can epistemic injustices be addressed [11,12]. On the one hand, these injustices prevent researchers from expressing themselves according to the conventions of scientific communication (testimonial injustice) and thus from participating fully in scientific discourse. Knöchelmann [13] calls this active participation general accessibility—as opposed to narrow accessibility, which includes only consumptive, passive access to scholarly literature—an essential part of the democratization of science. And, of course, science itself also benefits from a large plurality of voices, as otherwise discourses are unnecessarily narrow and biased.

Nevertheless, circumventing publication barriers is not an easy process for authors. Scholarly journals do not provide services^1^ that allow authors to bypass the editorial and peer review process, nor publishing in a journal. There are repositories to which one can gain access to the publication function with a minimum of know-how. However, with the aforementioned journals it is usually impossible. It is often stated that the existing reputation mechanisms within academia are far from perfect. Nevertheless, it is frequently the case that such mechanisms require researchers to publish in established outlets, namely academic journals. This makes the removal or at least the alleviation of barriers to OA publishing for authors essential for an epistemically just scientific system.

Given the comprehensive and exploratory nature of this review, the definition of OA encompassed the following provision mechanisms, commonly referred to as OA routes: gold OA, green OA, hybrid OA and diamond OA. These routes exhibit several distinctions, some of which relate to variations in their funding models, which directly influence authors, while others concern the type and extent of involvement required throughout the publication process. This study excludes black OA, bronze OA and delayed OA, as these OA subtypes are primarily defined by the reader’s access to the literature and, in the case of bronze OA, the rights of reuse [14,15]. Definitions of considered OA routes are given below.

Diamond OA—articles in free-to-access academic journals where both readers and authors do not pay subscription or article processing fees [16].
Gold OA—articles published in an open access journal, with all content made immediately available on the journal’s website, and where authors are required to pay article processing charges [15,17].
Green OA—article published in a subscription journal made available to the public in an institutional or disciplinary open access repository [15].
Hybrid OA—articles published in subscription-based journals offer authors the option to make individual articles freely available immediately upon publication by paying an article processing charge [18].

Nevertheless, the review does not disaggregate barriers according to OA provision mechanisms. This is due to the lack of detailed context in opinion papers and the study’s objective to display broad-based barriers identified in the literature, which do not directly stream from OA provision mechanism, but are seen as obstacles by researchers.

### 1.1. Research questions

To find out what hurdles researchers face, we aim to answer the following research question:

What are the barriers hindering or preventing researchers to publish OA described in existing literature?

Which specific barriers are described in the literature? Are there structures or hidden clusters in these barriers? How can we classify them to make them easier to address? Which barriers are researched, which are just mentioned or assumed? The aim here is to develop a broader understanding of barriers that are experienced by researchers that try to publish OA to develop a general framework of OA barriers. By answering these questions we want to give an overview of existing barriers, the extent to which they have been empirically investigated, and provide an overview of research gaps for further research.

## 2. Material and methods

A literature review based on the Xiao & Watson [19] guidelines was conducted. The investigation proceeded through four consecutive phases: the identification of relevant studies, the screening and selection of eligible studies, the extraction of data from the included papers and, finally, the summarizing and presentation of the results.

### 2.1. Identification of relevant studies

In order to compile a comprehensive literature list, it was decided to conduct searches across multiple databases: Dimensions, Web of Science, Scopus and Open Access Tracking Project (OATP). OpenAlex, a bibliographic catalogue of scientific publications, was not used as it was still undergoing refinement at the time of the literature search, which limited its reliability for systematic analysis. Search strings were designed based on the keywords derived from research questions and their synonyms. Pilot testing and modifications of the search queries were conducted to ensure their precision and accuracy. All searches were conducted on 16 October 2023, focusing specifically on titles and keywords. The final queries details are presented in table 1.

**Table 1.** Search queries.

data source	query	result
Dimensions	‘open access’ AND (publish* OR publication*) AND (barrier* OR obstacle* OR hurdle* OR injustice* OR exclusive* OR inequalit* OR discriminat* OR challeng* OR struggl* OR threat*) AND (person* OR author* OR scholar* OR scientist OR researcher OR academic*) NOT (‘is an open access article’ OR ‘This is an open access’ OR predatory OR medica* OR aggregat* OR service* OR covid* OR ‘an introduction’ OR protocol* OR ‘artificial intelligence’ OR ‘research data’ OR DNA OR computation* OR clinical* OR disease* OR simulation* OR treatment OR ‘machine learning’ OR ‘book review’ OR ‘correction’ OR lesson* OR framework OR editorial OR ‘Introduction’ OR ‘Afterword’)	874
Web of Science	(TS=((‘open access’ AND (publish* OR publication) AND (barrier* OR obstacle* OR hurdle* OR injustice* OR exclusive* OR inequalit* OR discriminat* OR challeng* OR struggl* OR threat*)) AND (person* OR author* OR scholar* OR scientist OR researcher OR academic*) NOT (‘is an open access article’ OR ‘This is an open access’ OR ‘predatory’ OR medica* OR aggregat* OR service* OR covid* OR ‘an introduction’ OR protocol* OR ‘artificial intelligence’ OR ‘research data’ OR DNA OR computation* OR clinical* OR disease* OR simulation* OR treatment OR ‘machine learning’ OR ‘book review’ OR ‘correction’ OR lesson* OR framework OR editorial OR ‘Introduction’ OR ‘Afterword’)))	486
Scopus	TITLE-ABS-KEY ((‘open access’ AND publish* OR publication) AND (barrier* OR obstacles* OR hurdle* OR injustice* OR exclusive* OR inequalit* OR discriminat* OR challeng* OR struggl* OR threat*)) AND (person* OR author* OR scholar* OR scientist OR researcher OR academic*) AND NOT (‘is an open access article’ OR ‘This is an open access’ OR ‘predatory’ OR medica* OR aggregat* OR service* OR covid* OR ‘an introduction’ OR protocol* OR ‘artificial intelligence’ OR ‘research data’ OR dna OR computation* OR clinical* OR disease* OR simulation* OR treatment OR ‘machine learning’ OR ‘book review’ OR ‘correction’ OR lesson* OR framework OR editorial OR ‘Introduction’ OR ‘Afterword’)	204

Moreover, on 20 November 2023 a search was performed in the OATP, a crowdsourced social-tagging project with the objective of capturing news and comments on OA to research [20]. OATP enables contributions from anyone by allowing them to tag resources related to OA.

Open Access Tracking Project (OATP)—311 returned results

(#oa.obstacle* AND #oa.author*) OR (#oa.obstacle* AND (#oa.comment* OR #oa.article* OR#oa.editorial*)) NOT (#oa.french OR #oa.italian OR #oa.spanish)

In addition to the systematic search, an expert consultation yielded two additional articles that were included in the dataset.

### 2.2. Selection of eligible studies

The searches conducted in Dimensions, Web of Science and Scopus returned a total of 1564 papers. Before importing them into Zotero, duplicates were identified and removed based on DOIs and titles, which resulted in the removal of 366 papers. In addition, one retracted paper and one podcast were excluded. The OATP query returned an additional 311 works. Subsequently, 24 papers were excluded after cross-referencing with the main dataset to eliminate duplicates. Following this stage, the papers were imported into a shared Zotero library. A team of two reviewers then independently screened the papers based on title and abstract and documented their assessment decisions by adding the tags ‘Relevant/reviewer’s name’ and ‘Irrelevant/reviewer’s name’. In cases where a paper received conflicting tags, the reviewers discussed it until a consensus was achieved. The reasoning behind the categorization as ‘irrelevant’ was recorded in the Zotero notes section.

Studies were evaluated based on the following criteria:

— Papers addressing researchers’ perceptions, attitudes and awareness of OA publishing.
— Papers discussing open science practices, adoption, challenges that also address the OA aspect.
— Studies that provide authors’ perspectives on the challenges of OA publishing.
— Papers written in English and German.
— Papers focused on OA journal articles publishing and self-achieving.
— Studies carried out globally, on a regional, national or institutional level.
— Availability of full-text versions.
— Study is either a research article, review article, conference paper, opinion piece (commentary, editorial, letter to author, etc.), or a grey literature study.
— All study designs are eligible for consideration, including those that utilize quantitative, qualitative, mixed, etc. methods, as well as opinion pieces.

Following the initial stage of title and abstract screening, 1280 papers were deemed irrelevant, leaving 207 papers for full-text review. The second stage also involved two reviewers. The 207 papers were split between them for individual assessment. This resulted in 113 papers being included in the final review.

It is important to clarify that the definition of barriers to OA used in the study encompasses a range of challenges that may not be explicitly labelled as ‘barriers’ in the included papers. They nonetheless align conceptually with the notion of impediments to OA publishing or are portrayed as factors that discourage authors from publishing in OA venues. The terms that were used to identify barriers such as ‘challenge’, ‘hurdle’ and ‘obstacle’ are reflected in our search queries (see table 1).

We decided to include theoretical research as well as opinion pieces (commentaries, editorials, etc.), which are usually excluded from systematic reviews. Opinion pieces such as editorials, offer valuable insights, often providing a personal perspective on a problem, situating it within a broader context, or highlighting significant debates and controversies within the subject [21]. The exclusion of a substantial number of papers, due to methodological shortcomings, may introduce selection bias and consequently diminish the review findings [22,23]. Among the exclusion criteria were papers that examined barriers to OA adaptation from the perspective of libraries (subscriptions to the literature) and publishers (funding sustainable business models). Papers addressing barriers to publishing OA monographs, OA book chapters and other types of OA research outputs are out of the scope of this review and were not considered.

**Figure 1.**
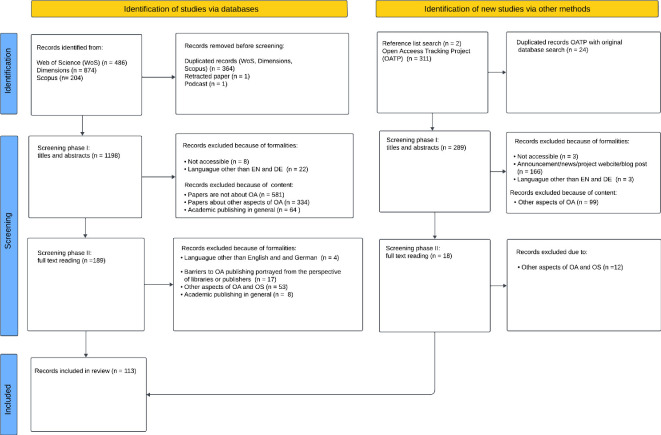
PRISMA-P flow diagram illustrating the screening phases.

The PRISMA-P [24] flowchart of the screening phases is presented in figure 1.

### 2.3. Data extraction

We used a collaborative Google Sheets spreadsheet for data extraction, adhering to the list of categories outlined in table 2. The data extraction process was conducted by two reviewers. As a consequence of the inclusion of non-empirical contributions in the selected papers, each identified barrier was assigned to a specific marker based on its depiction in the paper during the process of coding. If a barrier to OA publishing was the subject of an empirical study, or was identified as preventing researchers from publishing OA based on empirical study results, it was assigned the code ‘E’— empirical. If a barrier was discussed in an opinion paper or mentioned by an author in the introduction or literature review section of a paper, it was assigned the code ‘A’—appearance.

**Table 2.** Categories derived from the included studies.

heading	description
title of study	title of the article or study
author	author name
reference	full reference of the study containing a DOI or a URL
document type	research article, report, commentary, etc.
discipline	discipline or research field within which the research was conducted
method (if applicable)	research methodology (qualitative, quantitative, mixed, etc.)
geographical location	place where the study was conducted or referred to
year	year that the article was published
focus of study	overview of the aim of the study
barrier	what barrier to OA publishing was identified in the study. It also includes factors, challenges and various types of impediments that may not be explicitly labelled as barriers but nonetheless hinder authors from publishing in OA
barrier appearance in the paper	the way the barrier was mentioned in the paper. Was it an empirical study or was it simply mentioned?
key findings	conclusion of the study

### 2.4. Summarizing and results reporting

Once the table and analysis were completed, the results were summarized in the shared Google Document. In line with the study’s objective to develop a general framework of OA barriers, and given the inconsistent reporting of barriers related to specific OA provision mechanisms in the literature, barriers were not mapped specifically to OA types. Instead, the results are reported at the level of clusters and subclusters.

## 3. Results

### 3.1. Overview

**Figure 2.**
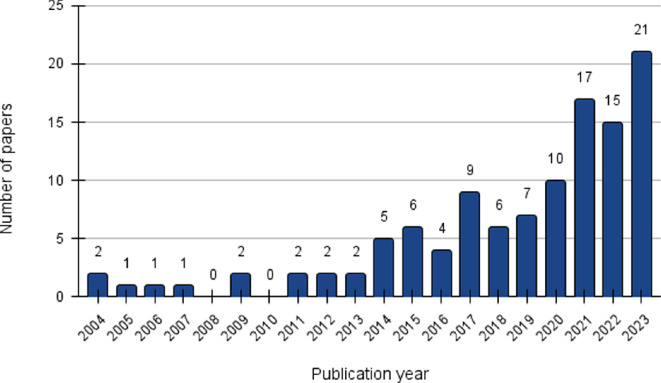
Annual distribution of papers included in the review from 2004 to 2023.

We included 113 papers in this review, spanning a period of 20 years, from 2004 to 2023 (figure 2). Although our search criteria did not specify a particular time limitation, the year 2004 was selected as the starting point due to the publication of the first paper addressing barriers to OA in the relevant literature. The data indicate significant variations in the number of papers published each year, reflecting a shifting interest in the subject. From 2004 to 2013, the number of publications was low and sporadic output, with no more than two publications per year. From 2014 to 2023, the number of publications increased, with the highest number of nine papers in 2017. From 2020 to 2023, there was a sharp rise, peaking at 21 papers in 2023.

Of the papers included in this study, 56.6% are research articles employing empirical methods. The remaining 43.4% represent non-empirical output, including theoretical works on the research subject, opinion pieces, commentaries, editorials, etc.

Geographically, 30.7% of papers focused on LMICs, while 24.6% had a global scope. Papers targeting European countries accounted for 10.5%, while those focusing on North America constituted 7%. Other countries were the subject of 3.5% of the papers and 23.7% of the papers did not specify a geographical focus.

**Figure 3.**
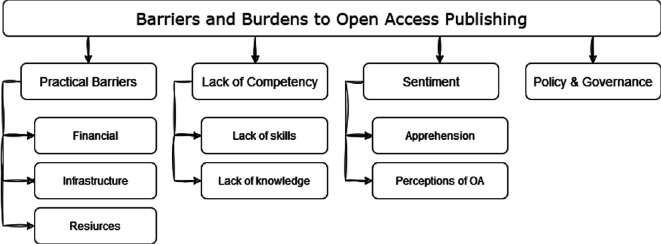
Hierarchical taxonomy for barriers and burdens to OA publishing for researchers identified in literature from 2004 to 2023.

A total of 82 distinct barriers were identified in the selected literature, which were subsequently grouped into four clusters and seven subclusters based on their thematic affinity (figure 3). The clusters and subclusters were developed inductively from the identified barriers. Two researchers independently reviewed the extracted barriers and grouped them based on thematic similarity through three rounds of team discussions. Each phase involved reviewing, refining and consolidating the clusters to enhance coherence and clarity. Although we did not perform formal intercoder reliability testing, consensus was reached through collaborative discussion and iterative refinement, ensuring the thematic structure was succinct and valid.

Despite the fact that some of the identified barriers do not appear to stem directly from OA provision models, we chose to include them to present a comprehensive account of the factors perceived and discussed as obstacles to OA publishing. Our aim is to highlight the complex and multilayered nature of the path towards achieving global OA, understood as the opportunity for authors to publish their work in OA outlets.

**Figure 4.**
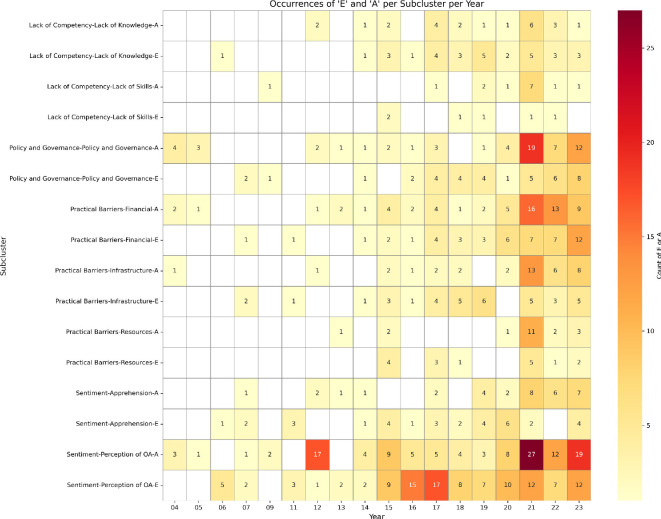
Heat map showing the occurrence of barriers to OA publishing from 2004 to 2023, categorized by subcluster and by the type of reporting: empirical ‘E’ or appearance ‘A’.

In order to capture the evolution of OA publishing barriers over time, we analysed the frequency with which each subcluster of barriers was reported annually across the 2004−2023 period. Figure 4 presents a heat map visualizing these occurrences, with barriers categorized by subcluster and distinguished by their mode of reporting: either empirical ‘E’ or anecdotal ‘A’.

These results indicate the presence of certain trends. For instance, discussions around financial barriers and policy and governance aspects have become increasingly frequent in recent years. Specifically, policy and governance aspects showcase a notable concentration of empirical reporting after 2018. Similarly, perception-related barriers to OA exhibit a sustained and growing presence across the entire time span, suggesting enduring ambivalence and ongoing debate surrounding the concept of OA itself. Infrastructure-related barriers, while not among the most frequently cited, have shown steady empirical reporting, especially between 2014 and 2020. This pattern may reflect growing recognition of the technical and organizational capacities required to support OA, particularly in less-resourced regions or institutions. Concerns related to lack of competency appear more sporadically and less intensively overall.

### 3.2. Practical barriers

The first cluster represents a set of tangible and concrete limitations, rooted in real-world constraints such as economic settings and logistical challenges, which effectively eliminate authors’ publishing options from the existing choices offered by the current scholarly communication system. This cluster encompasses 24.4% (*n* = 20) of all identified barriers, occurring in 102 papers (appendix A, table 12).

#### 3.2.1. Financial barriers

To the financial subcluster we assigned four barriers. Of these, the most prevalent obstacle was found to be unaffordable APC), as evidenced by their mention in 88 papers (table 3).

**Table 3.** Barriers to OA publishing within the Financial subcluster.

financial barriers			
barrier	occurrence	E	A
article processing charges	88	41	47
absence of funding	21	7	14
extra expenses for publishing	1	—	1
unaffordable conference participation fees	1	—	1

Financial barriers are predominantly rooted in the chronic scarcity or complete absence of research funding. This challenge is particularly acute in LMICs, where it is a systemic issue [25–28]. For instance, Druelinger & Ma [29] find that authors from LMICs contributed just over 1% of articles indexed in the Directory of Open Access Journals (DOAJ) in 2020. Funding scarcity is reported as a significant issue impacting researchers in various other contexts as well. These include (i) moderately affluent nations that are not classified among the world’s poorest 90 countries, but still experience significant limitations in research funding; (ii) institutions struggling with economic instability in post-Soviet regions [26]; and (iii) underfunded institutions in well-resourced countries such as the UK [26,30]. Evidence was also found within the field of political science where funded research was more likely to be published OA when compared with research output that was not financially supported [31].

In Rwanda, research funding is concentrated in a narrow range of disciplines with a strong focus on health, and neglects other disciplines [25]. The same paper mentions the significant influence of external funders, who often set the research priorities. This external control frequently overlooks the interests of local researchers and the specific needs of the country [25]. Shortages of funding in humanities and social sciences disciplines were reported in several cases [32–34].

According to Bonaccorso *et al.* [35], APCs can create substantial psychological barriers for authors, preventing them from submitting manuscripts to gold OA journals. Even when institutional support is available to cover fees, many researchers consider other financial demands to be of greater urgency, particularly those that directly facilitate their research endeavours. Limaye [36] argues that APCs may threaten research productivity. He analysed the finances of a research group at a major university and found that the annual costs of APCs could equal the salary of an additional full-time researcher or several part-time undergraduate student researchers. Segado-Boj *et al.* [37] concluded that authors perceive APCs more as a global threat to the advancement of science than as a personal deterrent to their own professional careers.

We also found that researchers outside conventional academic settings are highly affected by the author-pay OA model. In particular van Loon & van Loon [38] drives attention to healthcare workers, particularly for many self-employed physical therapists and other professionals in the rehabilitation field who conduct research, but are struggling to publish it OA. Additionally, Burchardt [39] identified additional categories of contributors to Danish scholarly journals who lack APC funding from institutions. These included retired scientists, unemployed individuals, students, self-employed individuals and professionals from public institutions.

Apart from high APCs, authors consider extra expenses like submission costs and colour figure printing fees to be impediments to OA publishing as well [25]. Limited mobility and the inability to participate in international conferences, which often invite authors to contribute to OA conference proceedings, also restrict the options of authors with regard to making their work freely available [40]. The reasons behind this include struggles with high registration fees, visa costs, accommodation expenses, etc.

#### 3.2.2. Infrastructure

The Infrastructure subcluster includes nine identified barriers (table 4). Of these, administrative burden, encompassing the efforts, time and resources required to meet the administrative and regulatory requirements for OA publishing, was extensively positioned as a hurdle to OA publishing. This was highlighted in 24 papers. Our findings indicate that when motivation of authors to publish OA meets with cognitive load due to extra tasks and requirements they tend to turn back to familiar publishing options [41]. One source highlighted that the administrative burden was especially relevant for hybrid journals [42]. Funded-gold payment systems, alongside the process of adding journal articles to the institutional repository, were also perceived by authors as ‘too much trouble’ to engage with [43,44]. One example that illustrates the frustration with self-archiving in institutional repositories was due to the fact that it is sometimes done via the research output management system with extensive metadata requirements [32]. Another reported case was related to the usage of author versions of articles [45]. Researchers reported that they may have become uncertain as to which version of an article they are permitted to self-archive. Finding the right version can be quite time-consuming, further complicating the self-archiving process. It was also identified, that the authors expressed reluctance to archive their work in a repository, citing concerns that the author’s version and the final published version, following peer review, might contain different reference lists [45].

**Table 4.** Barriers to OA publishing within the Infrastructure subcluster.

infrastructure			
barrier	occurrence	E	A
administrative burden	23	13	10
weak institutional infrastructure	19	4	15
few OA journals	10	5	5
technical barriers	9	5	4
practical barriers	5	2	3
speed of publication	5	4	1
lack of established subject repository	2	2	—
lack of metadata	1	1	—

A further common barrier reported in 22 papers was the weakness of institutional infrastructure. This included the absence of institutional repositories, the use of outdated software, insufficient information and communication technology (ICT), and inadequate IT support. Among the disincentives influencing authors’ choice of publication venue was the limited availability of OA journals, highlighted in 10 papers.

Lack of subject or discipline repositories was also identified as a hurdle [46,47]. Likewise, time from submission to publication was also cited as a factor that does not favour OA publishing [8,48–50].

#### 3.2.3. Lack of resources

Resource constraints were a central concern within the subcluster lack of resources (table 5). Notably, in six articles a lack of Internet connectivity or sufficient bandwidth was described as an impediment, limiting authors’ ability to effectively engage with OA publishing [51–57]. Additionally, four sources emphasized the disadvantageous position of institutions being unable to pay Internet service providers [54,58–60]. The related problem of the lack of computers with licensed research software was mentioned in two papers [56,60]. A wider issue was reported in four papers, namely the absence of reliable electricity as a barrier to OA publishing [54,56,57,60]. Raju *et al.* [61] noted that resource-scarce settings, in which many researchers operate in Africa, create a so-called challenge of ‘connectivity’, which hinders researchers from fully participating in scientific communication. In particular the African Continental Platform^2^ was referred to, which was launched to ‘de-northernize the publishing ecosystem’, particularly in the areas of accessibility, visibility and equity. However, the connectivity challenge hinders many researchers from being able to benefit from it.

**Table 5.** Barriers to OA publishing within the Resources subcluster.

resources			
barrier	occurrence	E	A
constraints on resources	14	4	10
lack of Internet access	6	4	2
lack of reliable electricity	4	2	2
high cost of Internet	4	3	1
no access to subscriptions of English-language literature	3	1	2
minimal research time	2	1	1
lack of access to a computer	2	1	1
inadequately stocked libraries	1	—	1

The inability for researchers to prepare high-quality manuscripts for OA journals because their institutions lack access to up-to-date English-language journals from major publishers was documented three times [60,62,63]. The same holds true for inadequately stocked libraries, which result in outdated research and, consequently, lead to the rejection of authors’ papers [59].

Minimal time for research was reported as a barrier to OA publishing in two papers. In Rwanda, for instance, heavy teaching loads and large class sizes reduced the time available for research. Additionally, many faculty members struggled with low salaries, which forced them to take on part-time jobs at other institutions. These challenges collectively make it difficult to conduct research and publish findings [60].

### 3.3. Lack of competency

This cluster includes all hurdles pertaining to the skill set and knowledge basis of researchers, or lack thereof, which impede their ability to publish OA. This might include lack of information on how to process, or lack of training on the option of OA publishing. Barriers from this cluster constituted 9% (*n* = 11) of all identified in the review and occurred in 47 papers (appendix A, table 13).

#### 3.3.1. Lack of skills

A total of 13 papers reported that language represents an obstacle to OA publishing, leading to a range of associated challenges (table 6). One of these hindrances was related to the overwhelming dominance of the English language in contemporary academic discourse, which limits the involvement of researchers from various parts of the world, and especially those from the Global South [64]. Some studies suggest that limited proficiency in English, rather than the intrinsic quality of the research, is a factor contributing to the rejection of manuscript submissions [59,65]. This is particularly relevant for countries where the official language is not English. A notable example is Kenya, which is home to 42 tribes with distinct languages, and Nigeria, which has approximately 371 tribes and over 521 languages [54]. The second challenge is that the majority of OA materials are predominantly written in English, which creates barriers for researchers to read, comprehend and effectively implement OA practices [54]. Another challenge emerges when translating indigenous knowledge into foreign languages, as this process risks losing both the meaning and the authenticity of the original content [59]. Raju *et al.* [61] emphasized the hurdles faced by non-English-speaking researchers in navigating dense legal content presented in English.

**Table 6.** Barriers to OA publishing within the Lack of skills subcluster.

lack of skills			
barrier	occurrence	E	A
language/linguistic	13	4	9
inadequate skills to publish OA	7	2	5

Cantrell & Collister [42] examined language from a different perspective and documented another barrier. They argue that information on OA, being presented online, is too complex. It is saturated with professional terminology, which leads to contradictory interpretations. The use of so-called ‘squishy language’, varied definitions, OA subtypes and licensing options results in a considerable cognitive burden. Furthermore, the plethora of decisions authors must make when pursuing OA publishing contributes to a status quo bias, leading some authors to view OA as an inferior alternative to subscription-based publishing.

Our review further revealed that insufficient skills for publishing OA was a common barrier. This was noted in seven studies. In particular, researchers encountered difficulties with specific technical proficiencies required, such as navigating specialized software, completing detailed metadata fields, editing, etc. [53]. Likewise, it was found that limited proficiency in broader digital literacy skills, including conducting effective Internet search, filtering information and navigating online sources is another impediment [46,54].

With regards to more specific publishing skills, we found that the complete absence or inadequate level of technological skills required to deposit in institutional repositories discourages researchers from considering this option [54].

#### 3.3.2. Lack of knowledge

We identified 14 papers that demonstrated that a lack of knowledge on how to publish in OA prevents authors from disseminating their work OA (table 7). One underlying factor that came out strongly was low awareness of OA publishing itself. Shashidhara & Sambathkumar [44] found in their study that many authors understand OA primarily as ‘free access’ to published work. However, there is a significant knowledge gap regarding the critical aspects of OA publishing such as funding mechanisms, the availability of OA journals, licensing requirements, etc. Additionally, in other instances OA was narrowly interpreted as synonymous with ‘gold’ or author-pays OA, overshadowing existing free-for-authors publishing models [66]. This limited perspective also contributes to a lack of awareness about the diamond publishing route [51].

**Table 7.** Barriers to OA publishing within the Lack of knowledge subcluster.

lack of knowledge			
barrier	occurrence	E	A
lack of knowledge about how to publish OA	14	10	4
lack of knowledge on how to deposit in a repository	13	7	6
low awareness of OA publishing	13	7	6
lack of sound information about OA	11	5	6
lack of transparency from publishers side regarding costs	1	—	1
lack of coordination among OA advocates	1	1	—
lack of clarity around the role of creative work in OA publishing	1	1	—

Apart from the fundamental aspects of OA publishing, low awareness at the institutional level also impedes participation in OA publishing. For example, in some instances researchers were unaware of whether their university had an institutional repository or a policy supporting OA [57,67]. They lacked knowledge about OA initiatives in which their own institution is involved, too. In another instance, researchers were unaware that an institutional repository has visibility outside of the university, and is indexed by Google Scholar [32]. Furthermore, researchers, particularly those from the social sciences and humanities, reported being unfamiliar with open subject-based repositories in their field [68]. Moreover, it was reported to be uncertain to some researchers if OA routes were permitted under their contracts [44].

Another related barrier highlighted in the literature was limited guidance and reliable information on OA publishing. Specifically, studies have shown that researchers lack resources and sound information regarding self-archiving [68]. A further study, conducted among researchers in educational technology, reported a lack of clear guidance on how to identify reputable OA journals within this particular field [69].

The lack of transparency with respect to submission costs and OA charges may discourage potential authors from engaging with OA journals, too [25]. The fragmented approach of OA advocates, with limited coordination, was also named as an obstacle to the widespread adoption of OA publishing [55].

Finally, we identified that ambiguity surrounding the dissemination of creative work acts as a deterrent for creative professionals from publishing OA [70]. Some practice-led researchers have expressed challenges in understanding how their creative contributions are treated within the OA framework, particularly in relation to exhibition opportunities and copyright concerns.

### 3.4. Sentiment

Under this cluster we grouped barriers that do not preclude the practical availability of OA alternatives from the choice set of authors. However, authors may be prevented from choosing certain venues for disseminating their research findings due to their personal beliefs. Those are perceived barriers. Sentiment represents the largest cluster, accounting for slightly more than half (51.2%, *n* = 42) of all identified obstacles in this review, occurring in 85 papers (table 8). It is split into two subclusters, namely ‘Apprehension’ and ‘Perception of OA publishing’. ‘Apprehension’ is defined as a negative sentiment (such as anxiety or fear) that can be experienced by researchers with regard to potential negative consequences in the future they may have to deal with if they publish OA. By contrast, the ‘Perception of OA publishing’ subcluster encompasses entrenched clichés of OA publishing, misconceptions and established traditions that are difficult to change. Literature with evidences of Sentiment barriers to OA publishing is presented in appendix A, table 14.

**Table 8.** Barriers to OA publishing within Apprehension subcluster.

apprehension			
barrier	occurrence	E	A
biases in scholarly communication industry	14	1	13
fear that paper will be misused or plagiarized	10	7	3
fear to violate publishers copyright policies	8	4	4
concerns for own career	8	4	4
reservation about preservation	6	3	3
perception that paying publication fees will mean less money available for research	3	1	2
concern that preprint leads to scooping	3	2	1
high APC can penalize research-intensive institutions by making them pay more	2	1	1
do not want to archive papers that have not been peer reviewed	2	2	—
supervisor/university advice against publishing OA	2	2	—
do not have permission from co-authors	2	2	—
uncertainty about future publishing environment	1	—	1
prejudice to diamond OA platform as it is a novice option that has not been tested sufficiently	1	—	1
open peer review may cause misunderstanding	1	1	—
fears for the standing of their members	1	—	1
concerns for career of their students	1	1	—
concern that preprint prevents publication	1	—	1
fear of violating state security measures	1	1	—

#### 3.4.1. Apprehension

The Apprehension subcluster comprises 18 identified barriers (table 8). Among these, conscious and unconscious biases in the scholarly communication industry are significant challenges faced by authors trying to publish OA. In particular, 14 papers emphasize the issue of geographical bias, driven by stereotypes in which research originating from Global North countries is often perceived as superior to that from Global South countries [29,34,52,60,65,71]. This bias contributes to high rejection rates of submissions from authors that submit their work from Africa [65]. One source highlighted another issue, where high-quality research by an African author on a pressing topic for the African continent, was rejected by a Global North journal because reviewers and editors deemed the topic outside their scope of interest [71]. Although the research was substantial, it was perceived as less relevant to Global North priorities, leading to the rejection of otherwise valuable scientific work. Another source discusses the importance of diversity among reviewers and editors of scientific journals, as their identity might influence what is published [72].

Plagiarism, misuse and potential exploitation of researchers’ work without adequate credit or attribution emerged as another commonly perceived barrier to OA publishing, mentioned in 10 papers. This apprehension surrounding plagiarism practices influenced authors’ choices both to publish in OA journals [33,73] and to deposit their work in repositories [74,75]. Similarly, concerns were expressed that publication of preprints may lead to scooping and stealing of ideas [49,68] or to preventing publication altogether [76].

In eight studies, researchers expressed concerns about unintentionally violating publishers’ copyright policies. These concerns were attributed to the complexity and ambiguity inherent in these policies [56,77]. Concerns regarding the advancement of researchers’ own careers (eight papers) and those of their students (one paper) also played a role in preventing researchers from choosing an OA venue. In two papers, supervisors were mentioned who advised their PhD students not to publish OA. The provided rationale was that OA publications are unnecessary for students who do not intend to follow academic careers after graduation [70] or, in the second case, that having only OA publications might negatively affect a student’s chances of securing academic employment [32]. Another deterrent was the apprehension about the preservation of OA content and the fear that OA works might be lost from the Web over time (six papers). This fear also applies to repository copies [74]. In one study researchers agreed that they would eagerly publish in OA journals if they provide a published copy of a journal [57].

Three papers report hesitancy to archive non-peer-reviewed papers due to concerns about the potential negative impact it might have on their professional reputation if the manuscript contains errors [45,74,78]. In two distinct studies, the lack of approval from co-authors for OA dissemination was identified as a contributing factor to choosing the venue [44,46].

One paper stated that authors may be inclined to avoid OA journals that employ an open peer review system due to its novice nature and its potential for significant confusion regarding its operational procedures [49]. Similarly, one paper indicated a prejudice against diamond OA platforms, considering them a novel option that has not been sufficiently tested and may not prove to be reliable [61].

Finally, one paper examining scientific communication in Libya identified state security as a barrier. The state’s policing and monitoring of the Internet have a significant impact on scholarly activities, with many researchers, particularly those studying sensitive topics, avoiding the publication of their work online due to concerns about being identified and subjected to interrogation [79].

#### 3.4.2. Perceptions of open access publishing

In the context of the Perception of OA subcluster, the perceived low quality of OA articles was identified as the most frequent deterrent for publishing OA, in 28 papers (table 9). Of the few sources that documented the origins of such assumptions, one reported a belief that OA operates under a ‘pay-to-play’ model, wherein subpar articles are allegedly accepted purely for financial gain [80]. Other associated issues that deter authors from publishing OA included the conviction that OA publications lack scholarly rigour (20 papers) and have inferior production standards (16 papers), such as inadequate copy editing and typesetting.

**Table 9.** Barriers to OA publishing within Perceptions of OA subcluster.

perceptions of OA publishing			
barrier	occurrence	E	A
low quality of papers	28	17	11
low prestige of OA publications	26	13	13
lower impact factor of OA journals	18	10	8
less scholarly rigour	20	11	9
low prestige of OA journals	19	10	9
predatory publishing	19	5	14
low production standards	16	7	9
cultural barriers	15	6	9
OA papers are cited less	11	5	6
negative perception towards OA, scepticism	11	5	6
low prestige of repositories	10	6	4
no incentives to publish OA	9	4	5
subscription publishing framed as status quo and OA is an alternative to ‘traditional publishing’	7	4	3
lower readership of OA papers	6	3	3
do not want to put my publication in OA	5	2	3
ideological barriers	3	2	1
different methodology traditions	1	—	1
did not equate OA publishing with practice-led research outputs	2	1	1
marketing	2	—	2
perception that knowledge should only be shared with readers that can understand it	1	—	1
industry-oriented research is incompatible with OA	1	—	1
behaviour reluctance: underestimation of or failure to see inaccessibility of closed access journals to the public	1	1	—
immediate access fosters fast and unreflecting consumption of literature	1	1	—
OA papers are primarily required for those pursuing an academic career	1	1	—

The perception that OA publications are less prestigious than their subscription-based counterparts was mentioned in 26 studies. For example, in one study researchers revealed that they associated OA papers with ‘grey literature’ and that to some extent they fear the ‘Wikipedia effect’ [77]. The term ‘Wikipedia effect’ draws a parallel between the possibility of rapid editing and publication of content on Wikipedia, which sometimes occurs without the rigorous oversight or peer review typical in academic contexts. The concern was that adopting a similar approach in a scholarly setting might compromise the reliability or integrity of the final output, as it bypasses traditional quality controls. Another example that reinforced this view was the ideology that ‘something that costs nothing is worth nothing’ [69]. In addition, 19 papers highlighted the low prestige of OA journals themselves, as opposed to the perceived lower prestige of OA papers mentioned above. One of the contributing factors to this perception is the lack of investment in marketing by OA journals, which results in a lack of recognition as a well-recognized brand [81,82]. Another aspect contributing to this view is the perceived lower impact factor of OA journals compared with subscription-based counterparts, which was discussed in 18 papers.

Similarly, the perceived issue of low prestige was relevant in the context of repositories. We found evidence to this in 10 papers. One study reported that the perceived low prestige of repositories was associated with the perceived low status of librarians, who typically manage institutional repositories. Consequently, the perceived lack of credibility of librarians in the eyes of researchers contributed to delays in submitting papers to the library [56].

The confusion of OA with predatory publishing is cited by 18 sources as a deterrent. Key characteristics of predatory OA journals, as identified by potential authors, include bypassing proper peer review procedures [83], publishing ‘as many papers as they can’ [84] and hidden fees [25].

Cultural barriers and research customs were identified as factors influencing the adoption of OA publishing in 15 papers. For instance, the deeply rooted tradition of disseminating knowledge through oral forms or as grey literature in African countries was noted [59]. One study identified a discrepancy between the research methods established in Rwanda and those required by international OA journals, portraying it as a tangible barrier to OA [60]. Ideological barriers were named in three papers. In 11 papers, it was found that researchers tend to avoid publishing in OA journals, driven by the belief that OA papers are cited less frequently. Another compelling reason for not choosing OA outlets was belief of its lower readership (six papers). Another reason for authors’ reluctance to embrace OA was a perception that knowledge should only be shared with readers who can fully understand and make use of it [32]. The incompatibility of industry-oriented research with OA publishing was identified as a barrier in one paper [70].

In one paper researchers expressed concerns that the rapid availability of scientific articles could diminish opportunities for in-depth analysis, meaningful engagement, and reflective discourse among peers. Over time, this was seen as potentially contributing to a decline in the overall quality of academic literature, a problem exacerbated by the increasing pressure to publish [77].

### 3.5. Policy and governance

The policy and governance cluster encompasses the impediments associated with the multifaceted nature of legal frameworks and regulatory practices within scholarly communication domains that impact the dissemination of research outputs. It covers policy and regulation frameworks of diverse groups of stakeholders included in knowledge creation and dissemination. This cluster accounted for 13.4% (*n* = 11) of the identified barriers addressed in 58 papers of those included in the review (appendix A, table 15).

The most frequently reported barrier in this category was legal issues, identified in 26 papers. Specifically, copyright retention was a prominent challenge, affecting both self-archiving in institutional repositories [45,68,75,81] and journal publishing [51,53] (table 10). Authors raised concerns about maintaining ownership of their intellectual property, emphasizing the importance of proper attribution and retaining control over the future use of their publications.

**Table 10.** Barriers to OA publishing within Policy and Governance cluster.

policy and governance barriers			
barrier	occurrence	E	A
legal barriers	26	11	15
lack of OA support from institution	16	7	9
academic reward system	15	6	9
OA journals are not indexed in preferable databases	10	3	7
inconsistent and non-transparent APC waiver policy	10	2	8
restrictive terms of APC waivers	8	2	5
lack of institutional policies	7	5	2
lack of government OA policy	6	1	5
lack of funder OA policy	6	2	4
lack of mentoring for early career researchers	3	—	3
prohibitions of the use of external storage devices like disks or flash drives	1	1	—

The lack of institutional support for OA was also identified as a barrier in 16 papers. One study underscored the importance of institutional support, suggesting that comprehensive policies, guidelines and operational frameworks are essential to establish a cohesive approach to OA [53]. Another study highlighted insufficient coordination between libraries and research or publication units as a barrier to effective deposition of papers in repositories [56]. Lack of institutional policies was reported as a barrier in seven papers. Related issues, such as absence of government OA and funder OA were mentioned in seven and two papers, respectively. A concrete example showcasing government influence, as we found in the literature, was related to Libyan state policies that promoted conventional publication over electronic publication [79].

The reluctance of researchers to publish OA was attributed to the failure of the research assessment system to recognize and reward such articles. This issue was highlighted in 15 papers. In Tanzania, for example, academic works published in OA journals charging APCs were explicitly deemed ineligible for academic promotion [53]. In Pakistan OA articles were not considered beneficial for grant acquisition [46]. It was also noted that contributions to institutional repositories did not count towards tenure or promotion, leaving researchers unmotivated to invest efforts in this task [56].

The prevailing practice of evaluating paper quality based on whether it is indexed by specific databases also contributes to this issue. We found evidence for this in 10 papers. Authors reported that they are hesitant to publish in OA journals that are not indexed by the preferred databases. For instance, one source noted that despite the large number of Indonesian journals listed in the DOAJ, the directory is not widely recognized within the country [85].

Inconsistent and nontransparent APC policies were reported as a hurdle in 10 papers. Specifically, authors mentioned poor communication of the availability of APC waivers and eligibility criteria from the publisher side. In one case, researchers pointed out that the decision on whether to provide a waiver is made by publishers rather than editors, leading to extended waiting periods for decisions [38]. Concerns also were raised regarding the considerable click depth required to access necessary information from the homepage. Building on the hurdles surrounding APC policies, it was noted that not all publishers offer a 100% waiver. In some cases, partial APC waivers result in publications still being financially unaffordable for authors. One case in Kenya described instances where the lack of APC waivers in many journals often forces authors to choose those that offer financial relief, which lead them to avoid more reputable journals and, as a result, limited the visibility and impact of their work [65].

Restrictive terms of APC waivers were indicated as an obstacle to OA publishing in eight papers. A specific example highlighted by Faciolince & Green [62] was that certain countries classified by the World Bank as LMICs, such as India and Nigeria, are often omitted from APC policies of publishers.

Other barriers identified in this cluster were the lack of mentorship for early career researchers [50,60,86] and the prohibition on the use of external storage devices, such as disks or flash drives [58].

### 3.6. Demographic factors

One important aspect we emphasize in this study is the role of demographic factors. Although these factors do not constitute barriers in themselves, they are frequently reported as influencing the likelihood of engaging in OA publishing. Three key factors were identified: academic status, gender and age. These factors appeared in a total of 24 papers (appendix A, table 16).

Gender was described as a factor influencing the likelihood to publish research output in OA in 12 sources (table 11). Specifically, four empirical studies reported that male researchers tend to publish in OA journals more frequently than their female colleagues [48,87–89]. This observed divergence can be explained by the differential impact on career prospects, where women appear to make a more cautious publication-choice investment, given the general gender productivity gap [87]. By contrast, only one source reported that female authors publish more in OA and are more likely to choose Gold OA than other OA subtypes [90]. Breuning & Akyol [31] and Lwoga & Questier [56] found that gender does not appear to directly influence the likelihood of publishing in OA. A consecutive study also by Breuning & Akyol [31], however, revealed that mixed gender and larger research teams have a higher likelihood of publishing in OA. Mahmood *et al.* [79] highlighted the challenges faced by women researchers in Libya, emphasizing the particular difficulties and constraints in scientific communication they encountered due to their gender.

**Table 11.** Demographic factors influencing OA publishing.

demographic factors			
item	occurrence	E	A
academic status	16	5	11
gender	12	6	6
age	8	2	6

In this context, Brabeck [91] drew attention to the additional cognitive and emotional labour that women and other marginalized groups often take on to ensure inclusivity in OA publishing disputes. She pointed out that the task of identifying who is excluded or disadvantaged by publishing policies and mandates should not be solely shouldered by members of these marginalized groups, yet it often is.

Instances where academic status was linked to OA publishing practices were identified in 15 sources. Among these, four empirical studies reported varying results: Migheli & Ramello [87] found that associate professors are more likely than other categories to submit their papers to OA; Hayman [50] observed that full professors are more likely to publish in OA; Ravikumar & Ramanan [73] reported that lecturers are more inclined to choose OA journals; and, finally, Tmava & Ryza [92] highlighted a stronger tendency for senior researchers to engage in OA publishing than others.

Age as a determinant of OA publishing was discussed in eight papers. Lwoga & Questier [56] and Tmava & Ryza [92] noted that senior faculty are more likely to participate in OA scholarly communications than younger ones. Asare-Nuamah [93] further highlighted a regional perspective, arguing that early career researchers in Africa are at a disadvantage in publishing OA due to their lack of well-established networks with peers outside the continent due to their young age.

## 4. Discussion

While the adoption of OA publishing has revealed various barriers and sparked ongoing discussions on how to overcome them, a comprehensive overview of the specific challenges faced by authors is still lacking. In this literature review, which includes 113 papers published between 2004 and 2023, we found ample instances describing barriers associated with OA publishing. The selection of papers covering a 20-year period revealed a sharp increase in the number of publications in recent years, particularly from 2020 onward. This trend suggests that efforts launched to advance OA following the Budapest OA Initiative have not been fully effective, as the topic remains a focal point of discussion. Another insight from the dataset is that 30.1% of the papers focused on LMICs, and suggests that disadvantaged socio-economic conditions exacerbate the challenges faced by researchers in these regions.

This review provides a broad bird’s-eye view on the landscape of barriers to OA publishing. Our intention is to illustrate the complex, multilayered nature of the journey towards achieving global OA, as the possibility to have one’s work published in an OA venue. In doing so, we aim to provide a general framework of barriers to OA. The literature review emphasizes that eliminating specific OA-provision barriers alone will not ensure equitable, diverse and fair opportunities to publish OA, as there are broader systemic issues to address.

Based on thematic affinity, we grouped 82 identified barriers into four overarching clusters: Practical Barriers, Lack of Competency, Sentiment, and Policy and Government. Previous influential works proposing the categorization of barriers include Xia [7], who called main barriers to OA publishing such as attitudes, awareness and action. Björk [81] introduced six categories: legal framework, IT infrastructure, business models, indexing services, academic reward system and marketing and critical mass. Anderson [94] suggested that barriers faced by authors can be characterized from three perspectives: ‘willing but unable’, ‘unwilling due to misunderstanding’ and ‘unwilling due to disagreement’. Nicholas *et al.* [95] cited publishing costs as the primary obstacle.

Contrary to our expectations, the Sentiment cluster turned out to be the largest in terms of identified barriers, accounting for 51.2% of the total number. These barriers reflect concerns that OA publishing may result in negative future outcomes for authors or be viewed as inferior to subscription-based journals, compounded by misconceptions and deeply ingrained traditions that are difficult to change. The most frequently reported ones were ‘low quality of papers’, followed by ‘low prestige of OA publications’. These barriers are intangible as they do not restrict authors’ access to the option of publishing OA. Instead, authors have this option available to them but may opt not to pursue it due to entrenched habits or deeply rooted prejudices. Some of them, such as the belief in the ‘low impact factor of OA journals’, can be easily verified and compared on a basis of journals, yet they still influence decisions not to submit to OA journals. It is difficult to trace the origins of these sentiments, as only a few papers shed some clarity on the reasons for these misconceptions, such as associations of OA publishing with grey literature, ‘Wikipedia effect’ or the belief that something that is free cannot be valuable.

Ranking second, the Practical Barriers subcluster accounted for 24.4% of the barriers, with high article processing fees being the most frequently reported barrier, occurring in 88 papers. Unaffordable APCs have been reported as a universal challenge, affecting researchers in both economically disadvantaged and affluent contexts. In these situations, the ability to cover fees primarily depended on the availability of funding. One of the current solutions to tackle the APC problem, particularly within the European landscape, is adaptation of diamond OA, which offers free publication for authors [96,97]. In this regard some publishers do their part too. An example is the Public Library of Science (PLoS), spending $2.5 million to solve ‘the lack of affordability—by thinking beyond the article and beyond the Article Processing Charge’ [98].

Administrative burden was the second most frequently identified barrier within this cluster, applying to both journal publishing and self-archiving in repositories. This issue is becoming increasingly recognized, as it underscores the growing workload associated with open research practices and administrative tasks. Without intervention, these escalating demands could lead to unsustainable expectations for academics, further straining their capacity to balance research with administrative responsibilities [99].

Legal barriers remain a serious drawback for researchers to publish OA, too. One of the key issues is rights retention, which deals with maintaining ownership of the author’s intellectual property. These concerns highlight the ongoing tension between the benefits of OA and the protection of authors’ rights. It is particularly relevant in light of the emergence of generative artificial intelligence practices and the growing partnerships with publishers that enable large language models to be trained on its authors’ content, not always providing authors the opportunity to opt out of such arrangements [100]. There is hope for positive changes in the gradual evolution of research evaluation, potentially bringing open research closer to the centre. Examples that are frequently cited in this context include the Coalition for the Advancement of Research Assessment (CoARA) [101], DORA [102], etc. Finally, a lack of competency was frequently identified as a significant barrier to OA, encompassing areas such as information literacy, technical skills and language proficiency.

Our review of the literature also revealed that certain barriers to open access publishing appeared to be location-specific. Lack of electricity and insufficient Internet bandwidth were identified as significant barriers that impeded authors’ ability to effectively engage with OA publishing. Another case is a strong censorship of a state, which compels researchers to publish in local, print-based outlets. Addressing these barriers requires targeted interventions, such as improving infrastructure, increasing investment in digital connectivity, and advocating for policy reforms to reduce state censorship and promote academic freedom.

Incorporating a temporal dimension into our analysis highlights the evolving nature of the OA publishing landscape. The rise of discussions of financial barriers and policy-related challenges post-2018, for example, may correspond with the emergence of new OA business models such as transformative agreements and implementation of Plan S. This temporal analysis underscores the need for context-sensitive responses to OA challenges, as some barriers appear to be time-bound or transitional, while others persist regardless of systemic change.

Demographic factors are oftentimes mentioned in the reviewed literature. While they are usually not a barrier in itself, they can have a significant influence on the prevalence of other barriers. In future research, these dimensions should always be recognized as an influential parameter. The findings show, for example, that gender, academic status and age play a roles in influencing OA practices. Gender differences seem to be shaped by general inequalities in academia, with some studies showing that male researchers more often publish in OA journals than women, while mixed-gender teams seem to prefer OA in comparison with more homogeneous ones. The role of women and marginalized groups in promoting OA as an additional responsibility on top of their work needs to be emphasized to understand the uptake of OA. An interplay with the other demographic factor identified in this study, the higher prevalence of researchers with higher academic status to publish OA, is likely, as such higher positions are still more often occupied by men. The influence of the age could be relevant when a personal network is needed to participate in OA publishing activities. The aforementioned patterns highlight potential structural reasons for lower OA uptake of researchers from marginalized groups, which need to be better understood and finally overcome.

### 4.1. Limitations of the study

Although this literature review contributes valuable insights to the field, it has several limitations that may affect the generalizability of the findings and should therefore be noted. Firstly, there is a language limitation, as only papers in English and German were included. The incorporation of papers in other languages such as Spanish and Portuguese would possibly have brought up different barriers or prevalences. This is key, since some barriers are location-specific. Examples for this include the lack of electricity and insufficient Internet bandwidth in African countries, or the fear of researchers not publishing in OA due to security concerns.

Secondly, despite querying four databases to gather the corpus, some relevant studies may have been omitted. In particular, during the query formulation phase, we noted that a number of papers with ‘open access’ in the title and/or keywords did not appear in the search results. Another category of potentially omitted studies includes those investigating researchers’ scholarly communication behaviours, which may address barriers to OA publishing as part of a broader analysis, but do not use the specific keywords included in our query. Additionally, there may be instances where unique barriers are published in journals that are not indexed in the selected databases. On the other hand, considering that this review is the first phase of a two-year study within the IDAHO project, the manageable number of papers included was necessary given the time span of the project [103].

Thirdly, our decision to include non-empirical papers in the review needs to be considered when judging the results, as these papers provide subjective perspective and lack the empirical rigour of primary research. However, this inclusion is necessary and important to answer the research questions.

We also acknowledge that the choice to report barriers in the papers using ‘E’ for empirical and ‘A’ for mentions has its shortcomings. Specifically, ‘E’ does not always mean that there was dedicated in-depth research on this particular barrier, but that it was reported as a finding of an empirical investigation. Since we did not track citations, one barrier cited in multiple papers could be counted as separate mentions, increasing the frequency of occurrence. In many cases, it would be worthwhile to investigate the barriers deeper.

The decision to include barriers that were mentioned but not necessarily empirically proven was a conscious one in order to build a more comprehensive spectrum of barriers. Without mapping barriers indicated purely as ‘A’ we would have run the risk of missing out on potential barriers for OA authors, an important factor since it was established in this study that even perceived barriers can affect OA publication. Including said barriers, thus, opens up future research to investigate if these barriers truly exist or are solely perceived by some authors.

Lastly, this review does not disaggregate barriers according to OA provision mechanisms (e.g. gold, green, hybrid, diamond). We acknowledge that this may oversimplify important contextual differences between OA types. This limitation reflects the inconsistent level of detail provided in the literature, particularly in opinion papers.

### 4.2. Directions for future research

Future research would benefit from a more granular analysis that explicitly maps barriers to distinct OA provision mechanisms.

Specific directions can be easily found with a look at the tables in appendix A. There are various barriers that appeared (‘A’) in a paper, but were not or not sufficiently empirically (‘E’) researched. We emphasize some examples. Although there are various publications on the Sentiment cluster, there is little empirical research on why easily refutable claims are still prevalent. Köster [41] can serve as an example of a study that dives into the psychological mechanisms behind these beliefs. Another under-researched topic is researchers outside academia: unaffiliated or independent researchers, researchers in industry, government agencies or from non-profit organizations, citizen scientists and others. Furthermore, biases against research from the Global South are often mentioned, but rarely researched. We need to understand better why studies on local crops are being desk-rejected by journals [71] to foster a more equitable and epistemically just academic communication.

## 5. Conclusions

Despite decades of advocacy, open access publishing remains fraught with systemic inefficiencies and inequities that extend far beyond those generated solely by OA provision mechanisms. High article processing charges continue to act as a gatekeeper, disproportionately excluding researchers from active participation in the global processes of knowledge production and dissemination. This financial barrier is compounded by sentiment-driven obstacles, such as scepticism about the quality of OA publications and fears that publishing OA might negatively impact academic careers. These issues highlight the entrenched norms of academia that resist change, even in the face of growing global demands for equitable access to knowledge.

The OA landscape is constantly evolving, but by examining how it has operated and identifying the barriers it has created, we can better anticipate future challenges. Understanding these patterns is critical to addressing the systemic flaws that risk turning OA into a mechanism of privilege rather than a tool for democratizing science. Tangible progress requires bold reforms, from rethinking financial models to challenging the cultural perceptions that sustain these barriers.

## Data Availability

The supplementary data for this study, covering the period 2004–2023, are available through the Leibniz Data Manager web platform [104].

## Footnotes

^1^
Think. Check. Submit? https://thinkchecksubmit.org/.

^2^
https://lib.uct.ac.za/openaccess/services/continental-platform.

## Appendix A

See tables 12–16.

**Table 12.** Literature presenting evidence on barriers to OA publishing within the Practical Barriers cluster.

financial barriers subcluster	
barrier	literature
article processing charges	Ali *et al.* [84]; Alizon [105]; Anderson [106]; Asare-Nuamah [93]; Baffy *et al.* [107]; Baro & Eze [57]; Bashorun *et al.* [54]; Batterbury [78]; Beasley [108]; Berteaux [82]; Björk [81]; Bonaccorso *et al.* [35]; Bosah [51]; Breuning & Akyol [31]; Bryans [109]; Burchardt [39]; Chakravorty *et al.* [83]; Cole *et al.* [86]; Cox & Abbott [60]; Cox [60]; David *et al.* [53]; De-Filippo [67]; Demeter [110]; Dhar *et al.* [111]; Druelinger & Ma [29]; Eve [33]; Faciolince & Green [62]; Farida *et al.* [85]; Floyd *et al.* [112]; Fox & Hanlon [59]; Frank [28]; Gadagkar [113]; Getz *et al.* [77]; Gownaris *et al.* [66]; Gray [114]; Gross & Ryan [70]; Hayman [50]; Hegde [115]; Hrynaszkiewicz [116]; Johnson [117]; Joseph [118]; Kenney & Warden [119]; Klebel & Ross-Hellauer [120]; Knöchelmann [13]; Lam & Langer-Gould [121]; Landis *et al.* [72]; Limaye [36]; Lwoga & Questier [56]; MoChridhe [64]; Mwanzu [65]; Neville & Crampsie [47]; Nguyen *et al.* [89]; Nosek & Bar-Anan [122]; OA in Physics [123]; Ollé *et al.* [124]; Peekhaus [125]; Peterson *et al.* [26]; Pinfield [126]; Powell *et al.* [127]; Quigley [32]; Raju *et al.* [61]; Ravikumar & Ramanan [73]; Rodrigues *et al.* [128]; Rosenblum *et al.* [34]; Ross-Hellauer *et al.* [30]; Santhanam & Goyal [27]; Scherlen [63]; Scott *et al.* [80]; Segado‐Boj *et al.* [37]; Seguya *et al.* [129]; Sengupta [40]; Severin *et al.* [8]; Shashidhara & Sambathkumar [44]; Sheikh [46]; Shockey *et al.* [55]; Siler *et al.* [130]; Sotudeh & Ghasempour [131]; Strömberg *et al.* [132]; Strømme *et al.* [88]; Tella & Onyancha [58]; Vacek & Kaliaperumal [25]; van Loon & van Loon [38]; Veríssimo *et al.* [133]; Vervoort & Bookholane [134]; Vuong *et al.* [135]; Vuong *et al.* [136]; Warlick & Vaughan [48]; Zulu & Twum-Darko [137]
absence of funding	Baffy *et al.* [107]; Bashorun *et al.* [54]; Bryans [109]; Cox & Abbott [60]; David *et al.* [53]; Druelinger & Ma [29]; Eve [33]; Faciolince & Green [62]; Frank [28]; Getz *et al.* [77]; Gross & Ryan [70]; Lam & Langer-Gould [121]; Peterson *et al.* [26]; Quigley [32]; Rosenblum *et al.* [34]; Santhanam & Goyal [27]; Scott *et al.* [80]; Sengupta [40]; Tmava & Ryza [92]; Vacek & Kaliaperumal [25]; Vervoort & Bookholane [134]
extra expenses for publishing	Vacek & Kaliaperumal [25]
unaffordable conference participation fees	Sengupta [40]
**infrastructure subcluster**	
administrative burden	Bryans [109]; Cantrell & Collister [42]; Cox [52]; Cox & Abbott [60]; Gross & Ryan [70]; Gunasekera [74]; Hadad & Aharony [68]; Johnson [43]; Klebel & Ross-Hellauer [120]; Köster *et al.* [41]; Lam & Langer-Gould [121]; Landis *et al.* [72]; Lwoga & Questier [56]; Neville & Crampsie [47]; Nosek & Bar-Anan [122]; Quigley [32]; Ross-Hellauer *et al.* [30]; Rumsey [138]; Shashidhara & Sambathkumar [44]; Shockey *et al.* [55]; Shukla & Khan [75]; Vacek & Kaliaperumal [25]; Vervoort & Bookholane [134]
weak institutional infrastructure	Asare-Nuamah [93]; Baro & Eze [57]; Bashorun *et al.* [54]; Bosah [51]; Brabeck [91]; Bryans [109]; Chakravorty *et al.* [83]; Cox [52]; Cox & Abbott [60]; David *et al.* [53]; Frank [28]; Knöchelmann [13]; Landis *et al.* [72]; Lwoga & Questier [56]; Rosenblum *et al.* [34]; Sengupta [40]; Sheikh [46]; Shockey *et al.* [55]; Zulu & Twum-Darko [137]
few OA journals	Baro & Eze [57]; Gownaris *et al.* [66]; Hrynaszkiewicz [116]; Köster *et al.* [41]; Lam & Langer-Gould [121]; Quigley [32]; Severin *et al.* [8]; Shockey *et al.* [55]; Vacek & Kaliaperumal [25]; Warlick & Vaughan [48]
technical barriers	Bashorun [54]; Björk [81]; Cox & Abbott [60]; David *et al*. [53]; Fox & Hanlon [59]; Joseph [118]; Mahmood *et al*. [79]; Raju *et al*. [61]; Tmava & Ryza [92]
practical barriers	Anderson [94]; Bashorun *et al.* [54]; Johnson [117]; Johnson [43]; Raju *et al.* [61]
speed of publication	Baro & Eze [57]; Hayman [50]; Severin *et al.* [8]; Warlick & Vaughan [48]; Zarghani *et al.* [49]
lack of established subject repository	Neville & Crampsie [47]; Sheikh [46]
lack of metadata	Shockey *et al.* [55]
**resources subcluster**	
constraints on resources	Baro & Eze [57]; Cox [52]; Cox & Abbott [60]; Faciolince & Green [62]; Fox & Hanlon [59]; Frank [28]; Landis *et al.* [72]; Klebel & Ross-Hellauer [120]; Lam & Langer-Gould [121]; Peterson *et al.* [26]; Raju *et al.* [61]; Rosenblum *et al.* [34]; Sengupta [40]
lack of Internet access	Bashorun *et al.* [54]; Bosah [51]; Cox & Abbott [60]; David *et al.* [53]; Lwoga & Questier [56]; Shockey *et al.* [55]
lack of reliable electricity	Baro & Eze [57]; Bashorun *et al.* [54]; Cox & Abbott [60]; Lwoga & Questier [56]
high cost of Internet	Bashorun *et al.* [54]; Cox & Abbott [60]; Fox & Hanlon [59]; Tella & Onyancha [58]
no access to subscriptions of English-language literature	Cox & Abbott [60]; Faciolince & Green [62]; Scherlen [63]
minimal research time	Bryans [109]; Cox & Abbott [60]
lack of access to a computer	Cox & Abbott [60]; Lwoga & Questier [56]
inadequately stocked libraries	Fox & Hanlon [59]

**Table 13.** Literature presenting evidence on barriers to OA publishing within the Lack of competency cluster.

lack of skills subcluster	
barrier	literature
language/linguistic	Bashorun *et al.* [54]; Cantrell & Collister [42]; Cox [52]; Cox & Abbott [60]; Fox & Hanlon [59]; Hedlund & Rabow [139]; Joseph [118]; MoChridhe [64]; Mwanzu [65]; Ross-Hellauer *et al.* [30]; Scherlen [63]; Sengupta [40]; Shockey *et al.* [55]
inadequate skills to publish OA	Bashorun *et al.* [54]; Bosah [51]; Brabeck [91]; Cox & Abbott [60]; David *et al.* [53]; Lwoga & Questier [56]; Sheikh [46]
**lack of knowledge subcluster**	
lack of knowledge about how to publish OA	Baro & Eze [57]; Bashorun *et al.* [54]; Brabeck [91]; Cox & Abbott [60]; David *et al.* [53]; Ghane [140]; Gownaris *et al.* [66]; Klebel & Ross-Hellauer [120]; Lwoga & Questier [56]; Nosek & Bar-Anan [122]; Perkins & Lowenthal [69]; Reinsfelder [141]; Shashidhara & Sambathkumar [44]; Shockey *et al.* [55]
lack of knowledge on how to deposit in a repository	Baro & Eze [57]; Bashorun *et al.* [54]; Bosah [51]; Cox & Abbott [60]; David *et al.* [53]; Gunasekera [74]; Lwoga & Questier [56]; Quigley [32]; Sheikh [46]; Shashidhara & Sambathkumar [44]; Siler *et al.* [130]; Shukla & Khan [75]; Zargaryan *et al.* [142]
low awareness of OA publishing	Anderson [94]; Bosah *et al.* [51]; Bryans [109]; Cole *et al.* [86]; Cox & Abbott [60]; De-Filippo [67]; Gross & Ryan [70]; Gunasekera [74]; Köster *et al.* [41]; Neville & Crampsie [47]; Quigley [32]; Scherlen [63]; Tmava & Ryza [92]
lack of sound information about OA	Anderson [94]; Bashorun *et al.* [54]; Bosah *et al.* [51]; Bryans [109]; David *et al.* [53]; Gross & Ryan [70]; Johnson [43]; Shashidhara & Sambathkumar [44]; Sheikh [46]; Zargaryan *et al.* [142]; Zhang [143]
lack of transparency from publishers side regarding costs	Vacek & Kaliaperumal [25]
lack of coordination among OA advocates	Shockey *et al.* [55]
lack of clarity around the role of creative work in OA publishing	Gross & Ryan [70]

**Table 14.** Literature presenting evidence on barriers to OA publishing within the Sentiment cluster.

apprehension subcluster	
barrier	literature
biases in scholarly communication industry	Brabeck [91]; Bryans [109]; Chakravorty *et al.* [83]; Cole *et al.* [86]; Cox [52]; Cox & Abbott [60]; Frank *et al.* [28]; Landis *et al.* [72]; Mwanzu [65]; Peterson *et al.* [26]; Raju & Badrudeen [71]; Raju *et al.* [61]; Rosenblum *et al.* [34]; Sengupta [40]
fear that paper will be misused or plagiarized	Bashorun *et al.* [54]; Eve [33]; Getz *et al.* [77]; Gunasekera [74]; Lwoga & Questier [56]; Quigley [32]; Ravikumar & Ramanan [73]; Shockey *et al.* [55]; Shukla & Khan [75]; Zargaryan *et al.* [142]
fear to violate publisher copyright policies	Brabeck [91]; Bryans [109]; Getz *et al.* [77]; Hadad & Aharony [68]; Hegde [115]; Köster *et al.* [41]; Lwoga & Questier [56]; Zulu & Twum-Darko [137]
concerns for own career	Brabeck [91]; Getz *et al.* [77]; Ghane [140]; Hayman [50]; Nosek & Bar-Anan [122]; Sarabipour *et al.* [76]; Severin *et al.* [8]; Warlick & Vaughan [48]
reservation about preservation	Eve [33]; Gunasekera [74]; Lwoga & Questier [56]; Ravikumar & Ramanan [73]; Sarabipour *et al.* [76]; Warlick & Vaughan [48]
perception that paying publication fees will mean less money available for research	Bonaccorso *et al.* [35]; Kenney & Warden [119]; Nosek & Bar-Anan [122]
concern that preprint leads to scooping	Hadad & Aharony [68]; Sarabipour *et al.* [76]; Zarghani *et al.* [49]
high APC can penalize research-intensive institutions by making them pay more	Bryans [109]; Kenney & Warden [119]
do not want to archive papers that have not been peer reviewed	Getz *et al.* [77]; Zargaryan *et al.* [142]
supervisor/university advice against publishing OA	Gross & Ryan [70]; Quigley [32]
do not have permission from co-authors	Shashidhara & Sambathkumar [44]; Sheikh [46]
uncertainty about future publishing environment	Johnson [43]
prejudice to diamond OA platform as it is a novice option that has not been tested sufficiently	Raju *et al.* [61]
open peer review may cause misunderstanding	Zarghani *et al.* [49]
fears for the standing of their members	Severin *et al.* [8]
concerns for career of their students	Warlick & Vaughan [48]
concern that preprint prevents publication	Sarabipour *et al.* [76]
fear of violating state security measures	Mahmood *et al.* [79]
**perceptions of OA publishing subcluster**	
low quality of papers	Alizon [105]; Baro & Eze [57]; Bosah *et al.* [51]; Chakravorty *et al.* [83]; Cox [52]; David *et al.* [53]; Getz *et al.* [77]; Ghane [140]; Gross & Ryan [70]; Hayman [50]; Hrynaszkiewicz [116]; Kangas & Hujala [144]; Kenney & Warden [119]; Lwoga & Questier [56]; Migheli & Ramello [87]; Perkins & Lowenthal [69]; Pinfield [126]; Quigley [32]; Ravikumar & Ramanan [73]; Reinsfelde [141]; Segado-Boj *et al.* [37]; Sengupta [40]; Severin *et al.* [8]; Shashidhara & Sambathkumar [44]; Shockey *et al.* [55]; Scott *et al.* [80]; Tella & Onyancha [58]; Xiao & Askin [145]
low prestige of OA publications	Ali *et al.* [84]; Anderson [106]; Baro & Eze [57]; Bonaccorso *et al.* [35]; Bosah *et al.* [51]; Brabeck [91]; Cox [52]; Cox & Abbott [60]; Farida *et al.* [85]; Getz *et al.* [77]; Gross & Ryan [70]; Gunasekera [74]; Hayman [50]; Hedlund & Rabow [139]; Köster *et al.* [41]; Nosek & Bar-Anan [122]; Perkins & Lowenthal [69]; Quigley [32]; Ravikumar & Ramanan [73]; Reinsfelder [141]; Ross-Hellauer *et al.* [30]; Severin *et al.* [8]; Strömberg *et al.* [132]; Tella & Onyancha [58]; Tmava & Ryza [92]; Warlick & Vaughan [48]
lower impact factor of OA journals	Ali *et al.* [84]; Baro & Eze [57]; Bonaccorso *et al.* [35]; Bosah *et al.* [51]; Cox [52]; Farida *et al.* [85]; Getz *et al.* [77]; Hayman [50]; Hegde [115]; Lam & Langer-Gould [121]; Lwoga & Questier [56]; Nosek & Bar-Anan [122]; Perkins & Lowenthal [69]; Reinsfelder [141]; Ross-Hellauer *et al.* [30]; Severin *et al.* [8]; Shockey *et al.* [55]; Warlick & Vaughan [48]
less scholarly rigour	Alizon [105]; Cox [52]; Ghane [140]; Gross & Ryan [70]; Hayman [50]; Hegde [118]; Kenney & Warden [119]; Lwoga & Questier [56]; Perkins & Lowenthal [69]; Pinfield [126]; Peekhaus [125]; Reinsfelder [141]; Scott *et al.* [80]; Segado-Boj *et al.* [37]; Sheikh [46]; Shukla & Khan [75]; Strömberg *et al.* [132]; Warlick & Vaughan [48]; Xiao & Askin [145]
low prestige of OA journals	Ali *et al.* [84]; Anderson [106]; Baro & Eze [57]; Bonaccorso *et al.* [35]; Bosah *et al.* [51]; Brabeck [91]; Cox [146]; Cox & Abbott [60]; Getz *et al.* [77]; Gross & Ryan [70]; Hayman [50]; Hedlund & Rabow [139]; Nosek & Bar-Anan [122]; Perkins & Lowenthal [69]; Ravikumar & Ramanan [73]; Reinsfelder [141]; Ross-Hellauer *et al.* [30]; Raju *et al.* [61]; Tella & Onyancha [58]
predatory publishing	Baffy *et al.* [107]; Brabeck [91]; Chakravorty *et al.* [83]; Cox & Abbott [60]; Eve [33]; Faciolince & Green [62]; Frank *et al.* [28]; Gadagkar [113]; Hrynaszkiewicz [116]; Lam & Langer-Gould [121]; Ollé *et al.* [124]; Quigley [32]; Santhanam & Goyal [27]; Scott *et al.* [80]; Sengupta [40]; Strömberg *et al.* [132]; Vacek & Kaliaperumal [25]; Vervoort & Bookholane [134]; Zhang [143]
low production standards	Baro & Eze [57]; Bosah *et al.* [51] Chakravorty *et al.* [83]; Cox [146]; Getz *et al.* [77]; Gross & Ryan [70]; Hayman [50]; Hrynaszkiewicz [116]; Klebel & Ross-Hellauer [120]; Knöchelmann [13]; Nosek & Bar-Anan [122]; Peekhaus [125]; Ravikumar & Ramanan [73]; Reinsfelder [141]; Sengupta [40]; Vacek & Kaliaperumal [25]
cultural barriers	Anderson [94]; Bashorun [54]; Breuning & Akyol [31]; Bryans [109]; Cox [146]; Cox & Abbott [60]; Eve [33]; Faciolince & Green [62]; Fox & Hanlon [59]; Johnson [117]; Johnson [43]; Joseph [118]; Mahmood *et al.* [79]; Nguyen *et al.* [89]; Raju & Badrudeen [71]
OA papers are cited less	Baro & Eze [57]; Brabeck [91]; Cox [146]; Getz *et al.* [77]; Ghane [140]; Hayman [50]; Hegde [115]; Nosek & Bar-Anan [122]; Raju *et al.* [61]; Reinsfelder [141]; Sengupta [40]
negative perception towards OA, scepticism	Baro & Eze [57]; David *et al.* [53]; Eve [33]; Getz *et al.* [77]; Hayman [50]; Nosek & Bar-Anan [122]; Perkins & Lowenthal [69]; Pinfield [126]; Reinsfelder [141]; Tmava & Ryza [92], 2023; Vuong *et al.* [136]
low prestige of repositories	Cox [52]; Farida *et al.* [85]; Hadad & Aharony [68]; Lwoga & Questier [56]; Raju *et al.* [61]; Sarabipour *et al.* [76]; Sheikh [46]; Shashidhara & Sambathkumar [44]; Tmava & Ryza [92]; Zargaryan *et al.* [142]
no incentives to publish OA	Cole *et al.* [86]; David *et al.* [53]; Faciolince & Green [62]; Gownaris *et al.* [66]; Nosek & Bar-Anan [122]; Quigley [32]; Reinsfelder [141]; Severin *et al.* [8]; Shockey *et al.* [55]
subscription publishing framed as status quo and OA is an alternative to ‘traditional publishing’	Brabeck [91]; Cantrell & Collister [42]; Ghane [140]; Gross & Ryan [70]; Köster *et al.* [41]; Perkins & Lowenthal [69]; Shockey *et al.* [55]
lower readership of OA papers	Baro & Eze [57]; Brabeck [91]; Cox [52]; Getz *et al.* [77]; Ghane [140]; Severin *et al.* [8]
do not want to put my publication in OA	Baro & Eze [57]; Eve [33]; Hayman [50]; Nosek & Bar-Anan [122]; Reinsfelder [141]
ideological barriers	Anderson [94]; Johnson [117]; Johnson [43]
different methodology traditions	Cox & Abbott [60]
did not equate OA publishing with practice-led research outputs	Gross & Ryan [70]; Quigley [32]
marketing	Berteaux [82]; Björk [81]
perception that knowledge should only be shared with readers that can understand it	Quigley [32]
industry-oriented research is incompatible with OA	Severin *et al.* [8]
behaviour reluctance: underestimation of or failure to see inaccessibility of closed access journals to the public	Köster *et al.* [41]
immediate access fosters fast and unreflecting consumption of literature	Getz *et al.* [77]
OA papers are primarily required for those pursuing an academic career	Gross & Ryan [70]

**Table 15.** Literature presenting evidence on barriers to OA publishing within the Policy and Governance cluster.

policy and governance	
barrier	literature
legal barriers	Anderson [106]; Bashorun *et al.* [54]; Berteaux [82]; Björk [81]; Bosah *et al.* [51]; Bryans [109]; David *et al.* [53]; Eve [33]; Getz *et al.* [77]; Gross & Ryan [70]; Hadad & Aharony [68]; Joseph [118]; Johnson [117]; Köster *et al.* [41]; Linde *et al.* [45]; Neville & Crampsie [47]; Reinsfelder [141]; Rumsey [138]; Sarabipour *et al.* [76]; Scott *et al.* [80]; Severin *et al.* [8]; Sheikh [46]; Shockey *et al.* [55]; Shukla & Khan [75]; Veríssimo *et al.* [133]; Warlick & Vaughan [48]
lack of OA support from institution	Baro & Eze [57]; Bashorun *et al.* [54]; Brabeck [91]; Cole *et al.* [86]; Cox [52]; Cox & Abbott [60]; David *et al.* [53]; Klebel & Ross-Hellauer [120]; Knöchelmann [13]; Raju *et al.* [61]; Rosenblum *et al.* [34]; Shockey *et al.* [55]; Sengupta [40]; Vacek & Kaliaperumal [25]; Warlick & Vaughan [48]; Zulu & Twum-Darko [137]
academic reward system	Baffy *et al.* [107]; Berteaux [82]; Björk [81]; Bosah *et al.* [51]; Brabeck [91]; Cole *et al.* [86]; Cox & Abbott [60]; David *et al.* [53]; De-Filippo [67]; Faciolince & Green [62]; Köster *et al.* [41]; Neville & Crampsie [47]; Quigley [32]; Sheikh [46]; Tmava & Ryza [92]
OA journals are not indexed in preferable databases	Berteaux [82]; Björk [81]; Brabeck [91]; Cox [52]; Cox & Abbott [60]; Farida *et al.* [85]; Hayman [50]; Perkins & Lowenthal [69]; Reinsfelder [141]; Santhanam & Goyal [27]
inconsistent and non-transparent APC waiver policy	Chakravorty *et al.* [83]; Druelinger & Ma [29]; Frank *et al.* [28]; Klebel & Ross-Hellauer [120]; Lam & Langer-Gould [121]; Powell *et al.* [127]; Santhanam & Goyal [27]; Vacek & Kaliaperumal [25]; Vervoort & Bookholane [134]; Zulu & Twum-Darko [137]
restrictive terms of APC waivers	Cox & Abbott [60]; Druelinger & Ma [29]; Faciolince & Green [62]; Lam & Langer-Gould [121]; Peterson *et al.* [26]; Ross-Hellauer *et al.* [30]; van Loon & van Loon [38]; Vervoort & Bookholane [134]
lack of institutional policies	Baro & Eze [57]; Bosah *et al.* [51]; Cox & Abbott [60]; David *et al.* [53]; De-Filippo [67]; Frank *et al.* [28]; Shockey *et al.* [55]
lack of government OA policy	Shockey *et al.* [55]; Zhang [143]
lack of funder OA policy	Bosah *et al.* [51]; Bryans [109]; David *et al.* [53]; Sengupta [40]; Shockey *et al.* [55,140]; Zhang [143]
lack of mentoring for early career researchers	Cole *et al.* [86]; Cox & Abbott [60]; Hayman [50]
prohibitions of the use of external storage devices like disks or flash drives	Tella & Onyancha [58]

**Table 16.** Demographic factors influencing engagement with OA publishing.

demographic factors	
item	literature
academic status	Asare-Nuamah [93]; Cole *et al.* [86]; Frank *et al.* [28]; Gross & Ryan [70]; Hayman [50]; Köster *et al.* [41]; Lam & Langer-Gould [121]; Lwoga & Questier [56]; Migheli & Ramello [87]; Ravikumar & Ramanan [73]; Ross-Hellauer *et al.* [30]; Santhanam & Goyal [27]; Sarabipour *et al.* [76]; Strömberg *et al.* [132]; Tmava & Ryza [92]; Vervoort & Bookholane [134]
gender	Asare-Nuamah [93]; Brabeck [91]; Breuning & Akyol [31]; Cole *et al.* [86]; Landis *et al.* [72]; Mahmood *et al.* [79]; Migheli & Ramello [87]; Ross-Hellauer *et al.* [30]; Scott *et al.* [80]; Strømme *et al.* [88]; Szymula & Simova [90]; Vuong *et al.* [136]
age	Asare-Nuamah [93]; Frank *et al.* [28]; Lam & Langer-Gould [121]; Landis *et al.* [72]; Mahmood *et al.* [79]; Lwoga & Questier [56]; Sarabipour *et al.* [76]; Tmava & Ryza [92]
